# A Tunable Metagratings Leaky-Wave Antenna Based on Liquid Crystal

**DOI:** 10.3390/s26072191

**Published:** 2026-04-01

**Authors:** Odai Hassan Raheem Al Soad, Kenneth Kalan John, Hanyi Fu, Jiahui Fu, Kuang Zhang

**Affiliations:** 1 School of Measurement-Control Technology and Communication Engineering, Harbin University of Science and Technology, Harbin 150080, China; kenneth.k.john1@gmail.com; 2 School of Electronic and Information Engineering, South China University of Technology, Guangzhou 510641, China; 202330410212@mail.scut.edu.cn; 3 School of Electronic and Information Engineering, Harbin Institute of Technology, Harbin 150001, China; fjh@hit.edu.cn (J.F.); zhangkuang@hit.edu.cn (K.Z.)

**Keywords:** leaky-wave antenna (LWA), metagratings (MGs), tunable beam steering, periodic structure, liquid crystal

## Abstract

An electrically tunable wide-beam-scanning metagratings leaky-wave antenna (MGs LWA) based on liquid crystal (LC) is proposed. Two-dimensional (2D) periodic slotted MGs with capacitive and inductive behaviors are etched on the bottom layer of the substrate and backed by a ground plane with an LWA framework. Two different slotted MG elements are adopted to suppress the open-stopband effects. A theoretical analysis is conducted to provide a conceptual framework for the equivalent electromagnetic fields generated by slotted MGs. Using LC, tunable beam scanning is achieved at a fixed frequency. The LC is placed between the inverted MGs LWA radiating metal and the ground plane to control the LC molecules’ orientation angle by applying a DC voltage across them, thereby adjusting the LC permittivity. Using the results obtained, the proposed antenna can be tuned up to 40° at a fixed frequency by applying a biased DC voltage ranging from 0 V to 10 V. The actual operating bandwidth is 40% for continuous beam scanning of 71°, with a scanned sensitivity of 8.35°/GHz at the zero voltage (*V* = 0 V), and beam scanning of 61°, with a scanned sensitivity of 7.17°/GHz at the saturation voltage (*V* = 10 V). The proposed MGs LWA has a realized gain of up to 13.84 dBi. Finally, the proposed antenna has excellent performance due to its potential to achieve wide tunable beam scanning with a narrow beamwidth compared to traditional LWAs’ limitation of radiation angle, depending on the excitation frequency, which makes the proposed antenna suitable in terms of range and sensing calibration for operation at a specific frequency in sensing communication and radar applications.

## 1. Introduction

With the rapid advancement of cutting-edge wireless communication technologies, there has been a rising need for efficient and flexible antennas. Leaky-wave antennas (LWAs) reveal excellent beam-scanning capabilities as a function of frequency variation, and they have a less complex architecture than traditional phased arrays, which depend on complex feeding networks and phase shifters [1]. However, with traditional LWAs, the beam direction is fundamentally dependent on frequency, limiting beam steering at a fixed working frequency. Thus, significant research on tunable LWAs has been conducted. Tunable LWAs may meet the needs of multi-scenario applications by allowing for adjustable beam scanning and frequency tuning under external control [2], which makes it easier to integrate them into multiple stations of a sensing network. Tunable LWAs have been advanced in recent years from frequency scanning to focusing more on controlling the phase constant (β) and leakage attenuation (α) at fixed operating frequencies. As a result, beam direction, beamwidth, polarization, and even multi-beam radiation can be adjusted. There are three basic types of adjustment: electrical tuning based on active device loading [3], dielectric/surface impedance tuning based on tunable media/materials (such as liquid crystals (LCs)) [4], and other switchable tuning methods [5].

Metagratings (MGs) are a subclass of electrically thin layer slabs that have recently been used to address the challenge of complex beam control in inhomogeneous metasurfaces with numerous degrees of freedom [6,7]. MGs were introduced as a novel idea for periodically planar arrays of artificially subwavelength meta-atom scattering particles that required only a single polarizable element for 100% conversion, permitting a set of propagating surface waves known as Floquet Modes (FMs) [8,9]. Furthermore, MGs generally comprise thin components (strips or slots) printed or etched onto a dielectric substrate and are characterized by low profiles, lightweightness, and low losses. This configuration allows a limited local FM that aligns with the demanding development required for propagation in a specific direction with nearly uniform efficiency under quasi-periodic boundary conditions. Novel metagratings-based leaky-wave antennas (MGs LWAs) are introduced to address the challenges of mutual coupling and open-stopband (OSB) effects that occur with traditional LWAs, while offering significant beam-scanning capabilities. These MGs LWAs consist of a parallel-plate waveguide including a periodic MG array etched/printed on the upper plate, located on a dielectric substrate with a PEC backing [10].

A recent technique for creating tunable beam-scanning LWAs at a fixed frequency utilizing LCs due to their adjustable permittivity by applying an electric or magnetic field [11,12], which operates at higher frequencies, has been presented. Furthermore, LC has an essential characteristic, namely, the capability of controlling farfield patterns based on its dielectric anisotropy, which is tuned by an external electric field. Tunable beam-scanning LWAs using LC have been proposed with a limited beam-scanning range [4,13], limiting the application concept of tunable farfield pattern LWAs using LC. Meanwhile, a proposed broadband microstrip LWA that uses LCs achieved a 32° beam-scanning range at 12 GHz [14]. Furthermore, a novel ⵣ-shaped microstrip LWA based on LC has been proposed to achieve tunable dual-beam radiation with an applied bias voltage of 0–20 V, which is suitable for satellite and radar communication systems [2]. Potential adjustment for fast beam control of LWAs is required nowadays in microwave and radar sensor technologies to provide dynamic target tracking in direction-of-arrival (DOA) estimation [15], remote vital sign monitoring [16], exact location identification [17], satellite and sensing communication [18], unmanned aerial vehicle (UAV) tracking systems [19], and vehicular radar applications [20]. A normal beam direction phase with frequency scanning is used in traditional leaky-wave antennas (LWAs), where the radiation angle depends on the excitation frequency. Although this approach is effective, it has the obvious limitation of changing the free-space wavenumber, which might affect the accuracy of the range and sensing calibration in systems which are configured to operate at a specific frequency.

**Figure 1:**
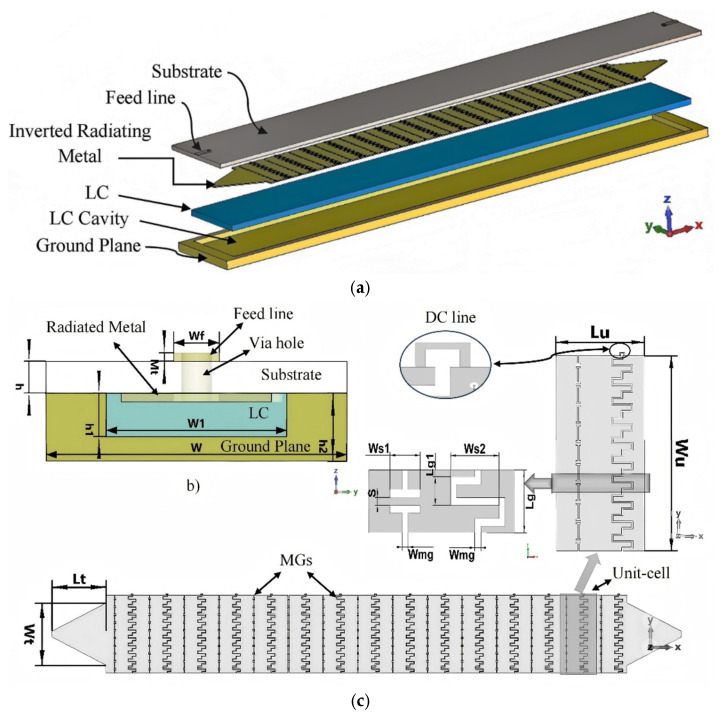
The structure of the MGs LWA. (**a**) Exploded view. (**b**) Cross-section view. (**c**) Bottom layer of the substrate (inverted radiating metal). *L_u_* = 9 mm, *W* = 25 mm, *W_u_* = 20 mm, *W_t_* = 16 mm, *L_t_* = 14 mm, *W_s_*_1_ = 0.5 mm, *W_s_*_2_ = 2 mm, *L*_g_ = 2 mm, *L*_g1_ = 0.667 mm, *W_mg_* = 0.15 mm, *S* = 0.2 mm, *W_f_* = 1.51 mm, *W*_1_ = 21 mm, *Mt* = 0.035 mm, *h* = 0.508 mm, *h*_1_ = 0.7 mm, and *h*_2_ = 1 mm.

In this contribution, tunable beam scanning for a slotted MGs LWA based on LC is presented, the system manufactured with PCB technology, as shown in Figure 1. In the framework of LWAs, periodic MGs are etched on a metal plate on the bottom of the dielectric substrate, revealing significant improvements. Slotted MGs provide capacitive and inductive behaviors. In order to optimize the degrees of freedom for matching impedance and minimizing the open-stopband effects, two individual periodic slotted MGs were employed for every unit cell. These slotted MGs produce equivalent electric and magnetic fields (magnetic currents), depending on the component state. For tunable beam scanning, the radiated plate (periodic MGs LWA) is placed as an inverted microstrip topology that can control the LC molecules’ orientation angle, which involves applying an external DC voltage through the radiated metal to change the LC dielectric properties from perpendicular permittivity ($E_{⊥}$ = 2.5) to parallel permittivity ($E_{∥}$ = 3.95). For the theoretical analysis, a theoretical model is developed and examined for electromagnetic fields produced by MGs, and the orientation angle of LC molecules is also examined. Finally, the proposed tunable MGs LWA measurement results are discussed.

The proposed MGs LWA based on LC is designed to be used as a tunable beam-scanning front-end component in electromagnetic sensing systems, especially in radar and directional wireless sensing. Tunable beam scanning of the observation area is needed in most sensing systems to determine which way the incoming or reflected wave is coming in or the way reflected signals are coming in in the case of target localization, environmental monitoring systems and UAV detection systems. Traditional LWAs normally operate on the principle of frequency scanning to steer the beam, a phenomenon that can result in poor sensing ability, since a change in frequency has a direct impact on both the range resolution and signal processing stability. By comparison, the proposed tunable MGs LWA provides beam steering that is voltage-controlled at a fixed operating frequency, enabling angular scanning without changing the spectrum of frequencies transmitted. This is made possible without losing the sensing bandwidth, along with directional interrogation of the environment. The range resolution and detection sensitivity of antennas are also important parameters of radar-based sensing systems, which are further improved by a narrow beamwidth and high realized gain. Thus, even though this contribution is a design study of antenna hardware and electromagnetic validation, the fixed frequency beam-scanning ability demonstrated offers a feasible front-end remedy for sensing architectures where electronically steerable radiation images in space are necessary.

## 2. Theoretical Analysis

### 2.1. Electromagnetic Fields

The proposed MGs LWA consists of a parallel-plate waveguide (inverted radiating metal and a ground plane), which is designed to cover the upper Ku-band and the full K-band areas (initially from 13.9 GHz to 27.2 GHz for zero-biased voltage). Two-dimensional periodic slotted MGs are etched on the dielectric substrate’s bottom layer, as illustrated in Figure 1b. The copper plate is positioned at *z* = −*h*_1_, and slotted MGs, which allow the input power to leak out, are positioned at *z* = 0. Permittivity (E), permeability (*µ*), and thickness (*h*_1_/2) are used to fill the dielectric medium at the half-plane. The length of *L*_u_ in the *x*-direction and the length of *L*_g_ in the *y*-direction are used to create a 2D periodic slotted MG array. A transmission line (TL) feeding system with a quasi-TEM polarized field at *x* = 0 excites the proposed MGs LWA. Using the method of moments (MoM), the electric fields on the slotted MGs (magnetic currents) are found to be equal to the fields, *E*_x_.

The total electric field in the *x*-direction from the slotted MGs is obtained in terms of magnetic currents due to a 2D periodic MG slot array at position (*x*, *y*, *z*) = (*pL*_u_, *qL*_g_, 0), *p*, *q* = 1, 2, 3 …, where *p* and *q* are the periodic MG slot arrays in the *x*-direction and *y*-direction, respectively. Therefore, the total electric field ($E_{x}^{t}$) can be expressed as

(1) $$E_{x}^{t}(x,y,z)=\frac{1}{L_{g}L_{u}}\sum_{p=-\infty }^{\infty }\sum_{q=-\infty }^{\infty }\left.\left(\begin{array}{l} {\tilde{G}}_{xy}^{-em}(K_{xp},K_{yq},z)\cdot {\tilde{M}}_{mgy}(K_{xp},K_{yq},z)\\ \cdot e^{-j(K_{xp}\cdot x+K_{yq}\cdot y-K_{z(p,q)}\cdot z)} \end{array}\right.\right)$$

where $K_{xp}=2\pi p/L_{u}$ and $K_{yq}=K_{0}\sin\theta_{in}+2\pi q/L_{g}$ are propagation constants of the *p*th, *q*th, and (*p*, *q*)th mode; ${\tilde{G}}_{xy}^{-em}$ is the spectrum domain of Green’s function that gives the *x*-directed electric field at *z* = 0; and ${\tilde{M}}_{mgy}$ is the Fourier transform of magnetic currents for MG slots in the *y*-direction.

Consequently, the radiated angle (*θ_n_* ≈ sin^−1^(*β*_n_/*K_z_*_(*p*,*q*)_)) determines the *n*th space harmonic phase constant (*β_n_*) for periodic MG slots (*β*_n_ = *β* + 2*nπ*/*L*_u_). In the fast-wave region (|*β*_n_| < *K_z_*_(*p*,*q*)_), a beam scan can radiate from the backward to the forward direction with an *n* = −1 space harmonic. When the main beam is scanned via the broadside, the open stopband typically appears. Two different periodic MG slots (MG1-type and MG2-type) are taken into consideration for a unit cell in order to suppress the open stopband. The open stopband is suppressed by adjusting the distance and the difference between the two periodic MG slots.

Green’s function, ${\tilde{G}}_{xy}^{-em}$, can be expressed based on Ohm’s law due to a set 1 V source at any point inside the LC cavity, *z* = −*h_d_*, as follows:

(2) $${\tilde{G}}_{xy}^{-em}(K_{xp},K_{yq},-h_{d})=-\frac{\sin\left(K_{z(p,q)}\cdot (-h_{d}+h_{1})\right)}{\sin(K_{z(p,q)}\cdot h_{1})}$$

The magnetic current calculation of the slotted MG aperture at location (0,0,0) is solved by defining the appropriate boundary condition. Depending on the location and size of the MG slot (MG1-type or MG2-type), the Fourier transform of magnetic currents on the MG slot can be determined by verifying the aperture integral equation using Galerkin’s approach as follows:

(3) $${\tilde{M}}_{mgy}=J_{0}\sum_{n=1}^{N=2}\left(An\left(K_{xp}\frac{Wsn}{2}\right)\left(\frac{n\pi L_{g}\left[e^{j(K_{yq}L_{g})}\cdot \cos(n\pi )-1\right]e^{-j(K_{yq}L_{g}/2)}}{{\left(K_{yq}L_{g}\right)}^{2}-{\left(n\pi \right)}^{2}}\right)\right)$$

where *J*_0_ is the current density, *W_sn_* is the width of the MG slots, and *A_n_* is the amplitude.

Note that the *L_g_* and *W_sn_* for each MG slot (*W_sn_* ⇨ *W_s_*_1_ for the MG1 type and *W_sn_* ⇨ *W_s_*_2_ for the MG2 type) determine Equation (3), which is the sum of the MG1-type and MG2-type Fourier transform magnetic currents.

### 2.2. Liquid Crystal Fundamentals

The ability of LC’s permittivity can be controlled by external DC voltages. When the applied voltage exceeds the Freedericksz threshold point, LC molecules start to reorient in the direction of the voltage fields, resulting in changing permittivities, known as perpendicular permittivity ($\varepsilon_{⊥}$) and parallel permittivity ($\varepsilon_{∥}$), or refractive indices of LC (∆*n* = √ $\varepsilon_{∥}$ − √ $\varepsilon_{⊥}$). Based on this idea, LC molecules were supposed to vary only in the z-direction (voltage field directions) with the orientation angle (*φ*(*z*)), and these molecules were treated as a unit vector, *n*^→^ = (*sinφ*, 0, cos*φ*).

In order to adjust the *φ*(*z*) for the LC molecules, an inverted MGs LWA is positioned in the bottom layer of the substrate. To verify that the electric field reaches the entire LC cavity, the bias voltage is connected to the inverted MGs LWA. The LC is housed in the LC cavity that is engraved in the ground plane. It is well known that LC molecules lie beside one another in the LC cavity as flattened ellipsoids. Each molecule’s *φ*(*z*) is dependent on both the Oseen–Frank energy (the energy given by splay, twist, and bend deformations for LC) and an external electric field. Consequently, by minimizing the Euler–Lagrange value, the generalized differential equation of *φ*(*z*) is obtained from the overall system’s energy as follows (see Appendix A for the detailed derivation):

(4) $$\frac{1}{2}{\left(\frac{V}{V_{th}}\right)}^{2}{\left(\frac{\pi }{h_{1}}\right)}^{2}\frac{\sin2\varphi }{\left(K_{11}\sin^{2}\varphi +K_{33}\cos^{2}\varphi \right)}-\frac{1}{2}\frac{\left(K_{11}-K_{33}\right)\sin2\varphi }{\left(K_{11}\sin^{2}\varphi +K_{33}\cos^{2}\varphi \right)}{\left(\frac{\partial \varphi }{\partial z}\right)}^{2}-\frac{\partial^{2}\varphi }{\partial z^{2}}=0$$

(5) $$V_{th}=\pi \sqrt{\frac{K_{11}}{\;E_{0}\;\Delta E_{r}}}$$

where *V*_*th*_ is the Freederieksz threshold voltage that induced no molecule reorientation.

∆*Ԑ*_r_ = *Ԑ*_r∥_ − *Ԑ*_r⊥_.

where *h*_1_ is the thickness of the LC and *K*_11_ and *K*_33_ are the splay and bend deformation elastic constants, respectively.

### 2.3. Sensing-Oriented Analysis

The performance of the proposed MGs LWA based on LC was investigated from the perspective of a traditional tunable antenna and its use in radar and microwave sensing systems. The antenna properties have a direct influence on the range resolution, angular discrimination, signal-to-noise-ratio (SNR), and phase stability in the case of integrated sensing systems (radar, satellite, monitoring, etc.). Therefore, this subsection provides a sensing-oriented analysis of the system’s phase behavior, beam steering dynamics, system-level sensing impact, and bandwidth operation. The biased voltage phase constant (*β*(*V*)) controls the radiation angle (*θ_n_*(*V*)) of the tunable MGs LWA structure as follows:

(6) $$\theta_{n}(V)=\sin^{-1}\left(\frac{\beta (V)+\frac{2n\pi }{L_{u}}}{K_{z(p,q)}}\right)$$

The proposed design also shows that the biased voltage can influence the effective permittivity of the liquid crystal layer ($E_{eff}$) as follows:

(7) $$\varepsilon_{eff}(V)=\varepsilon_{⊥}+\left(\varepsilon_{∥}-\varepsilon_{⊥}\right)\sin^{2}\left(\varphi (z)\right)$$

The proposed antenna just modifies the propagation constant and keeps a fixed operating frequency, which is essential for sensing systems. Additionally, the continuous voltage-controlled tuning reduced the inaccuracy of angular quantities and smoothed the tracking of objectives. The range resolution (∆R) of a radar system can be given as

(8) $$\Delta R=\frac{c}{2BW}$$

where *c* is the speed of light and *BW* is the proposed antenna bandwidth.

By adjusting the excitation frequency, beam scanning in a traditional LWA may result in variations in the calibration and accuracy of the range resolution. However, the proposed antenna maintains the wavenumber while tuning the beam scanning at a fixed frequency. As a result, the range resolution remains unchanged in the beam-scanning range.

The proposed tunable MGs LWA has the potential to offer valuable benefits to sensing systems where continuous beam scanning is needed, as in radar-based target localization and directional environmental sensing. Voltage control of beam scanning in a dynamic target tracking situation allows the range interrogation to proceed continuously without changing the operating frequency of the transmitter and, therefore, preserves range resolution stability. The narrow beamwidth and high realized gain of the antenna make the direction-of-arrival estimation more accurate. Moreover, the phase properties of the antenna are controlled by the propagation constant dispersion-controlled *β*, which changes predictably with the permittivity of the liquid crystal if a bias voltage is applied. This deterministic operation of phase behavior aids in coherent sensing operations that preserve the phase continuity along the radiating aperture, which is vital in radar imaging as well as coherent detection techniques. The continuous electrical tuning offered by the liquid crystal media as compared with switchable tuning methods based on discrete states (e.g., PIN diodes or RF switches) allows easier beam scanning with a higher resolution and eliminates sharp transitions between beams. This minimizes the effect of scanning quantization and facilitates the integration of systems by removing multi-scale switching networks. Thus, the proposed antenna design provides an electrically steerable front-end solution component for sensing systems which have stable frequency operation and high continuous scanning resolution requirements.

## 3. Design Considerations of Proposed MGs LWA

The proposed tunable MGs LWA consists of a dielectric substrate and a copper plate (ground plane) with thicknesses of *h* = 0.508 mm and *h*_2_ = 1 mm, respectively. The slotted MGs (arranged 10 times periodically for two different MGs in the *y*-direction) in the unit cell are periodically arranged 15 times with a distance of *L*_u_ ≈ 0.925*λ*_g_ in the *x*-direction and placed on the bottom layer of the dielectric substrate, as an inverted radiating metal, with a copper thickness of Mt = 0.035 mm. A Roger RT5880 dielectric substrate (Rogers Technologies (Suzhou) Co. Shenzhen Branch, Shenzhen, China) ($E_{r}$ = 2.2, tanδ = 0.0009) was selected. A two-port TL 50 Ω feeding system was used to obtain the excitation field for MGs LWA, a feedline (5 mm length and 1.51 mm width) located on the top layer of the dielectric substrate, and a tapered line (14 mm length, 1.51~16 mm width) located on the bottom layer of the dielectric substrate. A via hole connects the end of the feedline and the beginning of the tapered line through the dielectric substrate.

**Figure 2:**
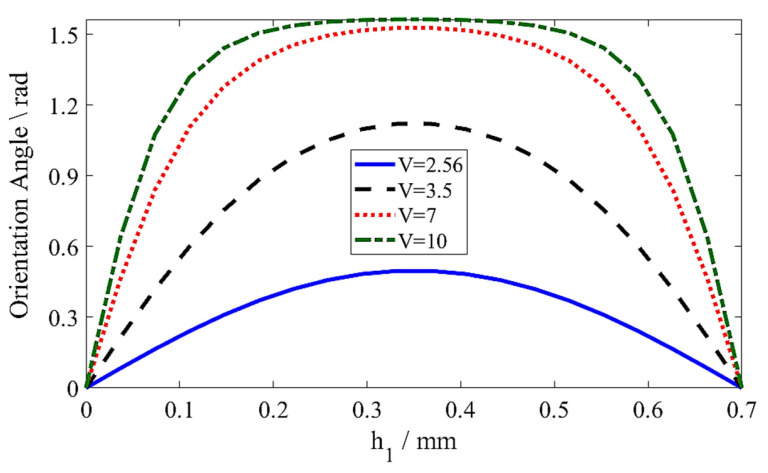
The calculated orientation angle versus the molecules’ position in the LC cavity for the different applied voltages.

LC molecules are perpendicular to the external electric field, *E* = [0, 0, *Ez*]. In this work, a commercial Liquid Crystal Mixture (QYPDLC-HD002-6, Qingdao QY Liquid Crystal Co., Qingdao, China) with a parallel permittivity of $E_{∥}$ = 3.95, tanδ = 0.022, and a perpendicular permittivity of $E_{⊥}$ = 2.5, tanδ = 0.012, was utilized. When there is no applied voltage (*V* = 0 V), the LC molecules are not actively reoriented by an external electric field, and the molecules have a highly disordered or weakly preferred orientation in the cavity, as no particular alignment has been overlaid on the surfaces., meaning that they are perpendicular to the directions of the electric field. After reaching a specific Fredericksz threshold value (*V* = 2.56 V) with 10% of the total LC molecules’ orientation, LC molecules started to reorient toward the electric field by progressively raising the applied voltage until they became parallel at the saturation voltage (*V* = 10 V) with 90% of the total LC molecules’ orientation, as shown in Figure 2. Equations (4) and (5) were used to calculate the orientation angle for the applied voltage, as shown in Figure 2.

**Figure 3:**
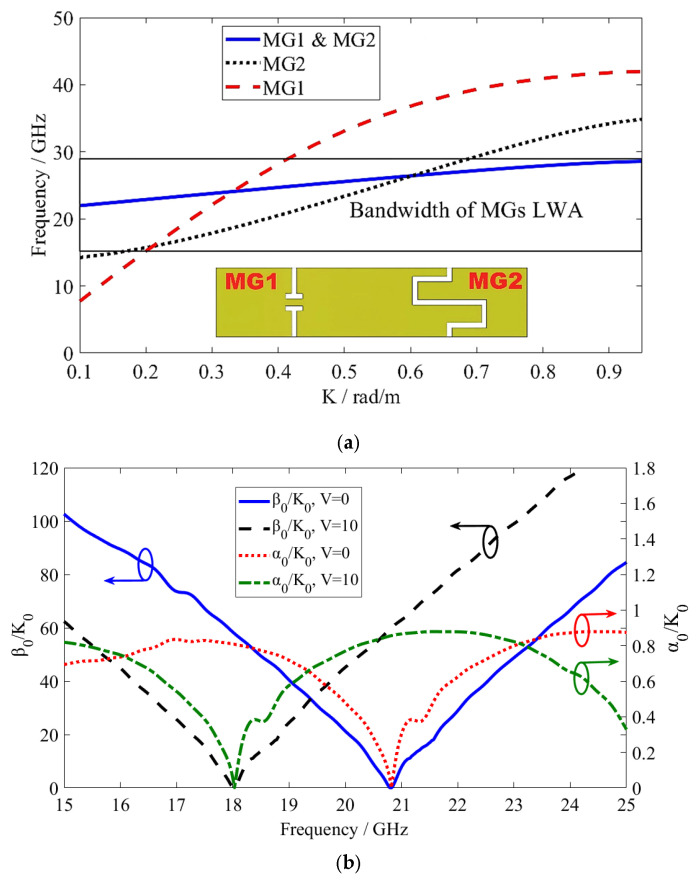
Calculated dispersion diagrams: (**a**) a single MG (MG1, MG2, and both); (**b**) a single MGs LWA unit cell (Blue/Black arrows represent the phase constant, Red/Green arrows represent the leakage constant).

The Eigenmode solver of the CST Studio Suite (v2025) was used to calculate the dispersion diagram shown in Figure 3. The calculation was performed by modeling a single MG1/MG2 in the CST Studio Suite to achieve a wide phase parameter. After that, the dispersion diagram for a MGs LWA unit cell was also calculated to determine the phase shift tunability. The Eigenmode solver calculation proceeds as follows: 1—choose the Eigenmode solver, 2—set up the boundaries of periodic MGs in the x- and y-directions, 3—set an initial value of 180 degrees for the phase parameter, 4—set the parameter sweep to configure the sweep of the phase parameter, and 5—define the required number of modes. The radiating Eigenmode associated with the leaky-wave propagation in the MG1 and MG2 periodic structures was chosen based on the field distributions across the operating bandwidth (13.9~27.2 GHz), the smooth dispersion behavior at the phase sweep, and the fast-wave radiation condition’s being met (|*β*_n_| < *K*_0_). Higher-order modes were eliminated.

Furthermore, the phase constant (*θ_n_* ≈ sin^−1^(*β*_n_/*K_z_*_(*p*,*q*)_)) determines the beam-scanning angle. The MGs LWA is defined by the complex propagation constant (*γ* = *α* + j*β*_n_), where *β*_n_ and *α* are the phase and leakage constants, respectively. As illustrated in Figure 3b, the continuous leaky-wave propagation occurs from the backward region (left-handed region) through the broadside (the transition frequency) to the forward region (right-handed region). The phase constant is shifted because of the change in LC permittivity caused by the voltage (0~10 V) applied to the radiated metal.

**Figure 4:**
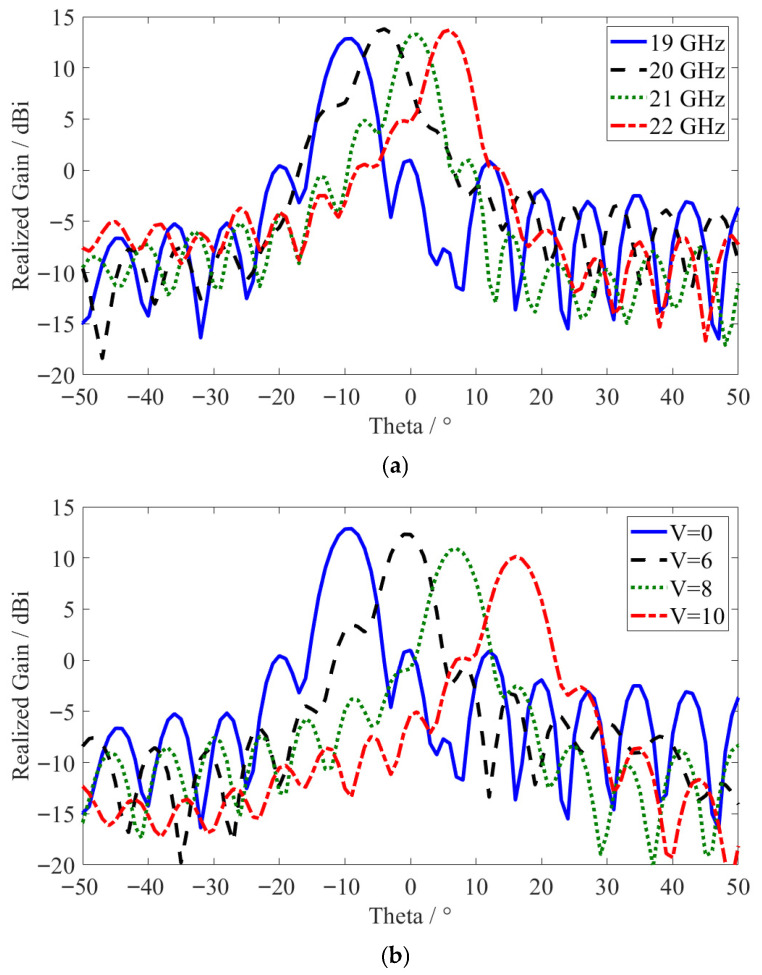
The simulated farfield radiation patterns across the broadside, 0°: (**a**) different frequencies in the zero-voltage case; (**b**) tunable beam scanning at 19 GHz.

In order to further confirm the open-stopband suppression around the broadside, other simulations were carried out to analyze radiation patterns in the frequency range of 19~22 GHz for the zero-voltage case and tunable beam scanning for different applied voltages at a frequency of 19 GHz, as shown in Figure 4. The results show that there was a continuous beam transition across 0° without splitting of the radiation pattern or sudden realized gain drop-offs, showing that there was no open-stopband behavior. Two metagrating setups (MG1 and MG2) vary the coupling between anti-propagating spatial harmonics, and this leads to less interaction to form the open stopband. As a result, the dispersion curve will be smooth over the broadside transition, which guarantees that the radiation performance will be stable and makes sure that the open-stopband effect is suppressed effectively.

## 4. Simulation and Measurement Analysis

**Figure 5:**
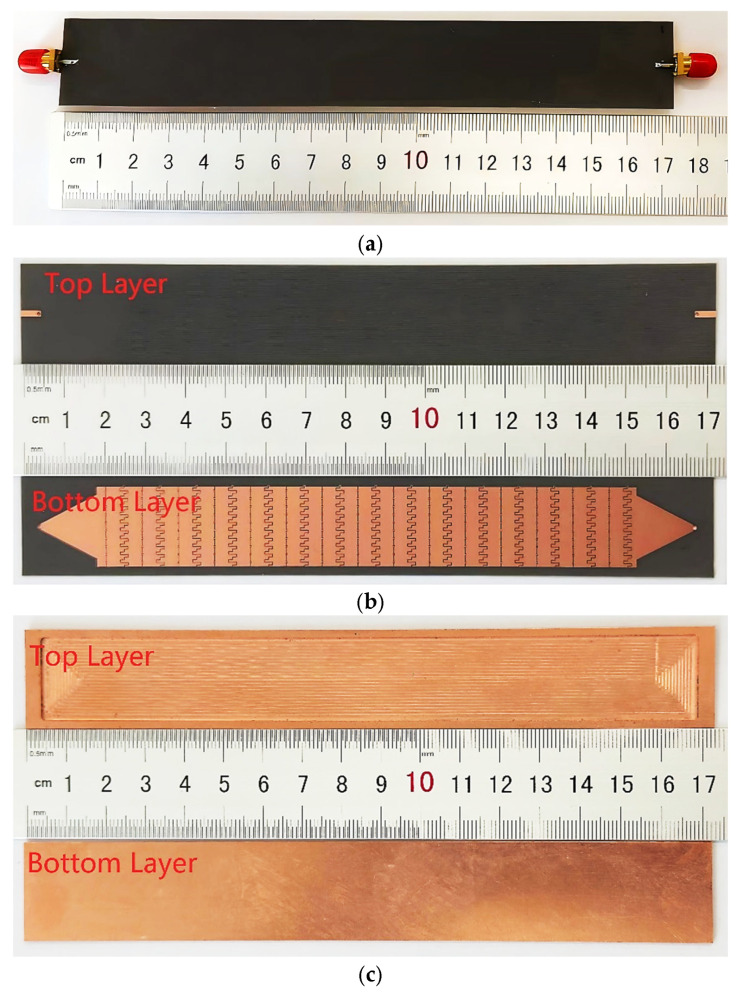
The fabricated prototype: (**a**) glued MGs LWA; (**b**) top substrate (top and bottom layers); (**c**) ground plane (top and bottom layers).

As can be seen in Figure 5, the MGs LWA was fabricated from a substrate and a copper plate with total dimensions of 173 mm × 25 mm × 1.508 mm, consisting of 15 unit cells with a length, *L*_u_ ≈ 0.925*λ*_g_ for each unit cell to enhance the transmission efficiency. The substrate was manufactured using Roger RT5880 dielectric material ($E_{r}$ = 2.2, tanδ = 0.0009) with a standard thickness of *h* = 0.508 mm. The radiating metal is located on the bottom layer of the substrate with a copper thickness of Mt = 0.035 mm, while the feedline is on the top layer of the substrate. A copper board with a thickness of *h*_2_ = 1 mm was utilized as a ground plane when drilling in the LC cavity with dimensions of 165 mm × 21 mm × 0.7 mm so that it could contain LC molecules. The MGs LWA was sealed with glue to avoid the LC leaking out. Two holes (LC in and LC out) were made on the copper plate to inject the LC into the LC cavity; these holes were soldered after the injection was completed.

**Figure 6:**
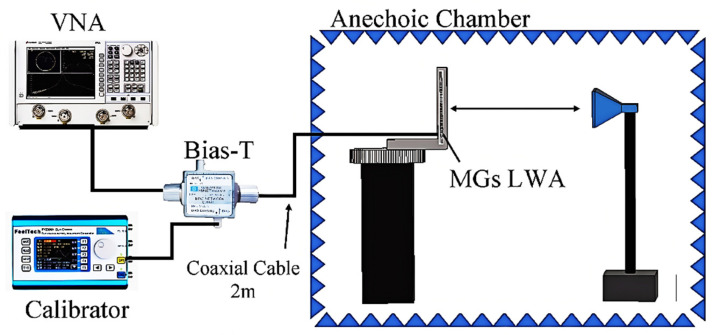
Sketch of the setup for the measurement process.

In order to measure and verify the performance, the proposed antenna was measured in a farfield anechoic chamber, as illustrated in Figure 6. The distance between the proposed antenna under test and the standard horn antenna was sufficient to meet the farfield requirement (*R* ≥ 2*D*^2^/*λ*), where *D* is the maximum size of the proposed antenna. The proposed antenna was attached to a rotating platform, while a calibrated standard horn antenna was taken as the reference antenna. The radiation patterns were measured by turning the rotating platform, and the gain was determined via the gain comparison method using the calibrated horn antenna under the same measurement conditions. A Vector Network Analyzer (VNA), the Keysight (Agilent Technologies Inc., Santa Clara, CA, USA) N5251A (10 MHz~110 GHz); a calibrator; a biasing network (Bias-T); 50 Ω coaxial cables (2 m in length); and a receiver were used to test the proposed MGs LWA. In order to excite the antenna via the SMA connector at port 1, the biasing network summed the DC voltage and RF signals. The DC voltage was applied by the calibrator between 0 and 10V constantly, considering that the MGs LWA copper plate was also connected to the DC voltage source’s ground.

**Figure 7:**
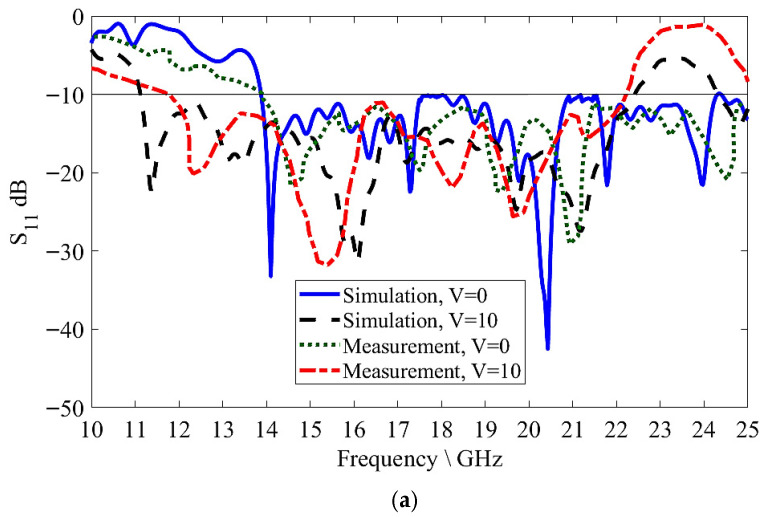

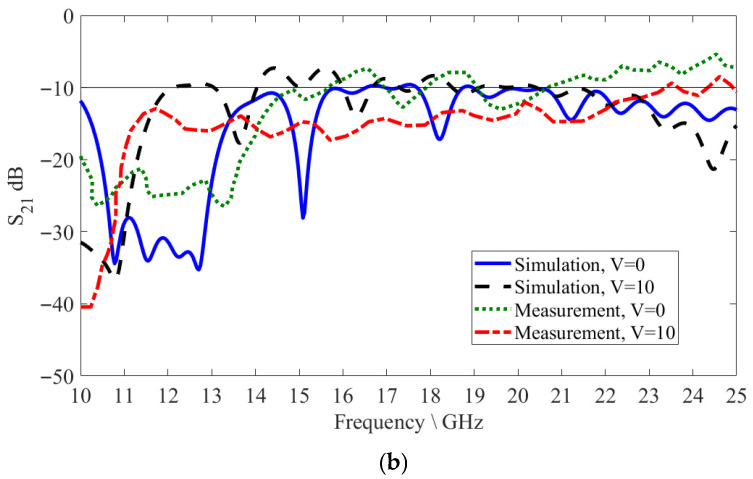
Adjustable S-parameters of MGs LWA: (**a**) S_11_; (**b**) S_21_.

Figure 7 presents the simulation and measurement results for the S-parameters. The applied DC voltage adjusted the LC dielectric permittivity, which determined the tunable bandwidth of the S-parameters. With a bandwidth of 13.9~25 GHz (53.3%) for the zero-voltage cases (*V* = 0 V) and 11.1~22.4 GHz (54%) for the saturation voltage cases (*V* = 10 V), S_11_ measured less than −10 dB, with a total actual operating bandwidth of 13.9~22.4 GHz (40% actual bandwidth) for all voltage cases, as shown in Figure 7a, indicating that the antenna achieved sufficient impedance matching at the input. Meanwhile, the S_21_ results were around −10 dB, which means that most of the input power radiated along the MGs LWA structure, as shown in Figure 7b. On the other hand, the glue layer between the substrate and the copper plate used to prevent LC leaking, the SMA welding, and the fabrication tolerance caused a slight mismatch between the simulated and measured results, which was not considered in the simulation process. Table 1 shows the effect of the unit cell number of the MGs LWA structure on the simulated radiation efficiency and transmission coefficient, S_21_, in the zero-voltage case. The radiated efficiency increased with the increase in the unit cell numbers, while S_21_ decreased. In order to achieve the most radiated input power and maintain the proposed antenna at the lowest size, a 15-unit cell structure was selected, which had an 85% efficiency and approximately −10 dB for S_21_. A high number of unit cells led to additional conductors and dielectric losses with an increase in the physical size of the proposed antenna.

**Figure 8:**
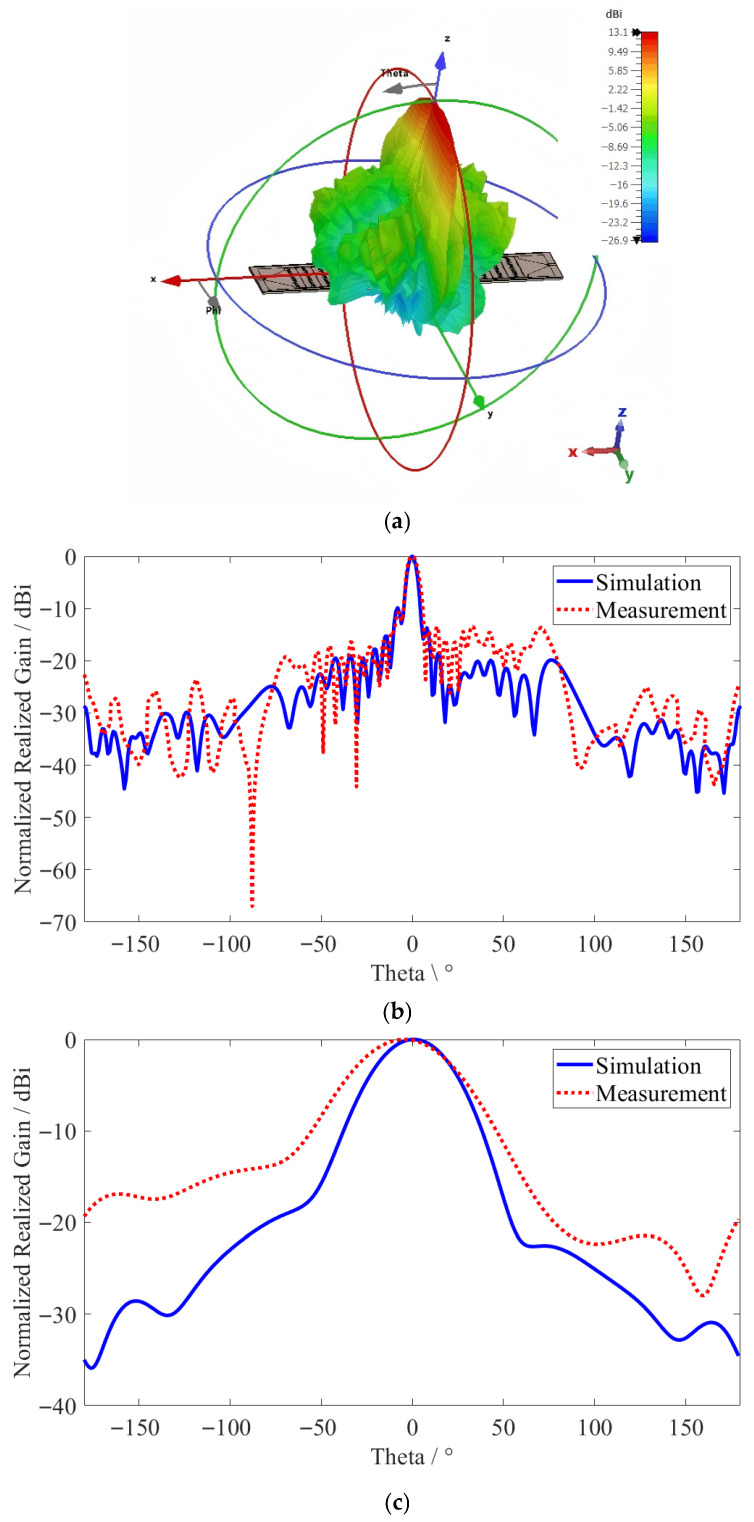
The farfield radiation pattern at 20.8 GHz: (**a**) 3D view; (**b**) E-plane; (**c**) H-plane.

Figure 8 represents the farfield radiation pattern at the center frequency of 20.8 GHz for the zero-voltage case, when the LC permittivity is perpendicular ($E_{⊥}$ = 2.5) to the DC voltage field. The Cartesian coordinates of the E-plane and H-plane, where the proposed antenna is fed by port 1 (forward direction), are shown in Figure 8b and Figure 8c, respectively. Given a −3dB half-power beamwidth (HPBW) of 5.2° in the E-plane and 41.7° in the H-plane, the main lobe direction angle is achieved at 0°, showing clear agreement between the simulation and measurement results.

**Figure 9:**
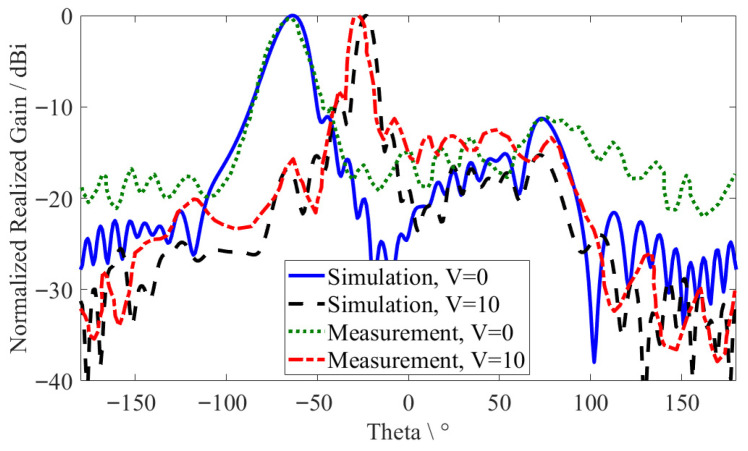
The simulated and measured E-plane farfield radiation patterns at 13.9 GHz.

**Figure 10:**
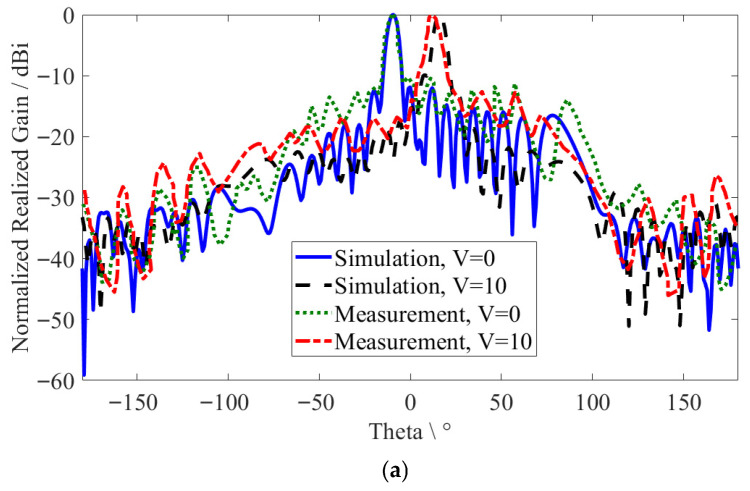

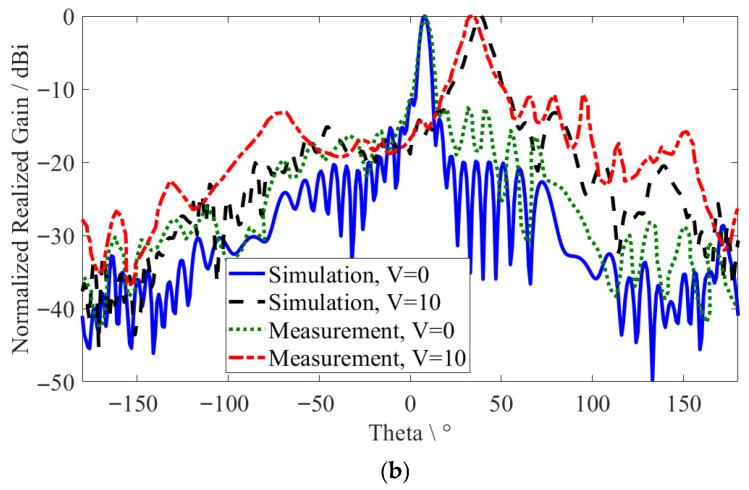
The simulated and measured E-plane farfield radiation patterns: (**a**) at 19 GHz; (**b**) at 22.4 GHz.

To continue the measurement process, the MGs LWA is fed by a bias network, which sums the RF signal with a bias DC voltage, and it is placed towards the receiver to measure the realized gain and tunable beam-scanning angles. At a fixed frequency of 13.9 GHz (the backward region), the proposed antenna is tuned up to 40° in the E-plane from (*θ* = −63°) with an HPBW of 18.4° at the zero voltage, *V* = 0 V to (*θ* = −23°), with an HPBW of 10.1° at the saturation voltage, *V* = 10 V, caused by an adjustment in the LC permittivity, as illustrated in Figure 9. At the same time, Figure 10 shows the proposed antenna tuning from *θ* = −9° with an HPBW of 6.2° for *V* = 0 V to *θ* = +16° with an HPBW of 6.5° for *V* = 10 V and from *θ* = +8° with an HPBW of 5.3° for *V* = 0 V to *θ* = +39° with an HPBW of 9.4° for *V* = 10 V at a fixed frequency of 19 GHz (broadside region) and 22.4 GHz (forward region). The measurement and simulation results showed good agreement. However, the small shift between the simulation and measurement results reached up to 3° for the *V* = 10 V case, which is acceptable, indicating that LC molecules were unable to be entirely oriented to the saturation case due to their physical characteristics and dead zones. (Dead zones are regions in an LC cavity (not under IMSL radiating metal) that have a lower electric field intensity caused by the electrode geometry and fringing-field effects, which lead to the partial reorientation of the LC molecules due to small changes in the LC permittivity as compared to the regions under greater intensity in the electric field (under IMSL radiating metal).) Furthermore, adjusting the LCs with the DC voltage may result in small-scale side effects, leading to results with limited reproducibility.

**Figure 11:**
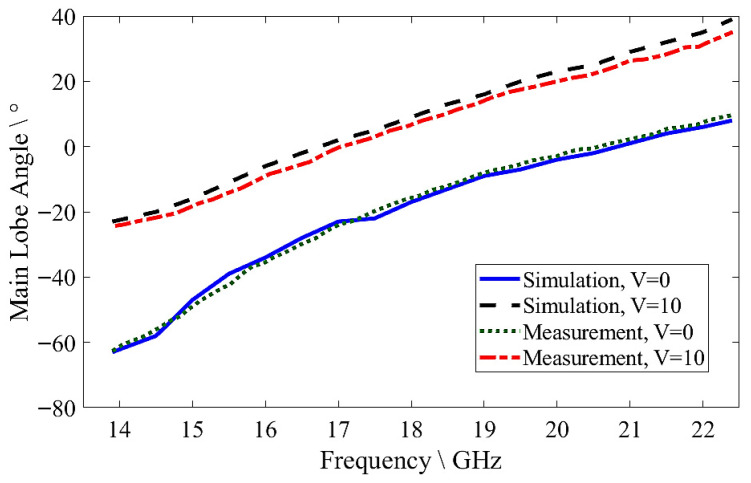
The MGs LWA main lobe directions.

According to the actual bandwidth frequencies of 13.9~22.4 GHz, Figure 11 represents the MGs LWA beam-scanning range in the E-plane for the zero-voltage case (*V* = 0 V) from −63° to +8°, with a scanning sensitivity of 8.35°/GHz, and for the saturation voltage case (*V* = 10 V) from −23° to +39°, with a scanning sensitivity of 7.17°/GHz. Note that the beam-scanning range was measured as the maximum value at actual bandwidth frequencies of 13.9~22.4 GHz with a scanning step size of 0.1 GHz.

**Figure 12:**
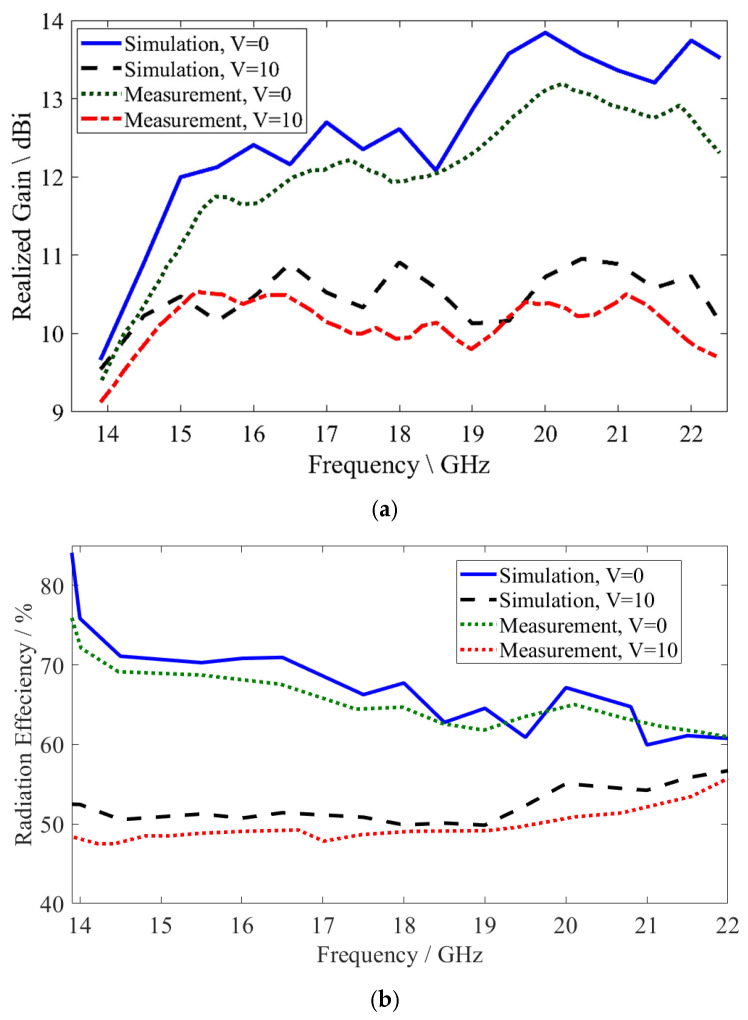
The simulated and measured results for MGs LWA: (**a**) realized gain; (**b**) radiation efficiency.

The simulated realized gain of the proposed MGs LWA was achieved from 9.66 to 13.84 dBi for *V* = 0 V and from 9.54 to 10.95 dBi for *V* = 10 V, while the measured results were obtained from 9.4 to 13.19 dBi for *V* = 0 V and from 9.12 to 10.53 dBi, as shown in Figure 12a. The realized gain was found to be in good agreement with the simulated and measured results. Additionally, the simulated radiation efficiency obtained reached up to 84% for *V* = 0 V and up to 56% for *V* = 10 V, while the measured results obtained reached up to 75.9% for *V* = 0 V and up to 54.8% for *V* = 10 V, as shown in Figure 12b. The radiation efficiency drops with the increase in the biased voltage due to the influence of the LC loss tangent and bias-dependent dielectric losses. The measurement results verified the efficiency of the proposed antenna, with slight differences between the simulated and measured results caused by the substrate’s being placed above the radiating metal, the SMA connectors, the glue layer, the fabrication tolerance, and/or error during the measurement process.

Table 2 shows a comparison of the proposed MGs LWA and other related works. In [2], a tunable dual-beam ⵣ-shaped microstrip LWA with a size of 157.5 mm × 36 mm × 2.129 mm for the X-band was proposed, based on nematic LC, and achieved a tuning range of 25° and a measured gain of up to 7 dBi, with a range resolution of up to 12.5 cm. A new substrate-integrated waveguide (SIW) with a microstrip line LWA was designed with a switchable LC (which is not capable of continuous tuning) as 56 split-ring resonators with a total size of 112 mm × 8.6 mm × 2.7 mm to obtain switchable beam scanning with up to a 12 dBi measured gain and a 1.5 cm range resolution, as presented in [13]. An LWA with a reconfigurable structure was presented to achieve a tunable beam-scanning range of 32° with a measured gain of 8.1 dBi and a 5 cm range resolution, as presented in [14]. In [21], a half-mode corrugated (HMCSIW) LWA with LC loaded to reach a 25° tunable range with a measured gain of up to 10 dBi was presented, while the range resolution reached up to 15 cm. Furthermore, the authors of [22] designed a decoupling structure for a 43-element LC-based holographic LWA to obtain switchable tuning for 20° intervals (which is not continuous) with a 12.6 dBi measured gain and a 5 cm range resolution. In this work, a tunable MGs LWA based on LC was designed with two periodic slotted MGs to suppress the open-stopband effects and to achieve wide tunable beam scanning of 40° with a high measured realized gain of up to 13.19 dBi, while the range resolution reached up to 1.76 cm. In addition, the proposed antenna and related works were compared with a 77 GHz automotive radar standard (typical bandwidth of up to 5 GHz) with a range resolution of 3 cm; the proposed antenna showed a better range resolution (1.76 cm < automotive radar standard). The range resolution is the theoretical range resolution that can be obtained when the antenna is incorporated into a radar sensing system as a front-end component that makes use of the entire bandwidth available to it. This contribution is devoted to antenna hardware design and electromagnetic validation; system-level radar implementation and experiments in radar target detection were not included in the framework of the current study.

## 5. Conclusions

A tunable beam-scanning metagratings leaky-wave antenna based on liquid crystal is presented. The radiating metal consisted of a 2D periodic array for two different slotted MGs, characterized by capacitive and inductive behaviors to suppress the open-stopband effects, etched on the bottom layer of the substrate and backed by a ground plane. A theoretical analysis determined a conceptual framework for the equivalent electromagnetic fields generated by the slotted MGs. A liquid crystal was placed between the radiating metal and the ground plane to achieve tunable beam scanning at a fixed frequency. The inverted MGs LWA controls the LC molecules’ orientation angle by applying a DC voltage through them, which results in adjustment of the LC permittivity. As a result, the proposed antenna is tuned from −63° for the zero voltage (*V* = 0 V) to −23° for the saturation voltage (*V* = 10 V) at a fixed frequency of 13.9 GHz. The actual operating bandwidth is 13.9~22.4 GHz for the beam-scanning range of −63°~+8° with a scanning sensitivity of 8.35°/GHz (*V* = 0 V) and −23°~+39° with a scanning sensitivity of 7.17°/GHz (*V* = 10 V). A realized gain of 9.54~13.84 dBi was obtained for all voltage cases. Finally, the MGs LWA achieved wide tunable beam scanning with a narrow beamwidth, which makes it suitable for sensing communication and radar applications.

## Figures and Tables

**Table 1:** The effect of the number of unit cells in the MGs LWA in terms of radiation efficiency and the S_21_ parameter.

Unit Cell No.	Radiation Efficiency (%)	S_21_ (dB)
2	10.7	−2.3
4	28.1	−3.7
6	46.8	−5
8	53.4	−6
10	66	−7.7
12	72.6	−8.1
15	85	−10
18	88	−12
21	90	−15.3
24	91	−16.9

**Table 2:** Comparison of the proposed antenna and related works.

Antenna	LWA Type	Dimensions (mm)	Actual Bandwidth (GHz)	Tunable at a Fixed Frequency (°)	Meas. Realized Gain (dBi)	Range Resolution (cm)	Compared to 77 GHz Radar Standard
In [2]	ⵣ-microstrip	157.5 × 36 × 2.129	9.3~10.5	25	7	12.5	poor
In [13]	SIW-ML	112 × 8.6 × 2.216	30~40	Switchable	12	1.5	better
In [14]	Microstrip waveguide	122 × 20 × 10.88	11~14	32	8.1	5	poor
In [21]	HMCSIW	160 × 25 × 1.1	10.2~11.2	25	10	15	poor
In [22]	LC-based holographic	258 × 15.4 × 2.51	9~12	Switchable	12.6	5	poor
This Work	Slotted MGs	173 × 25 × 1.508	13.9-22.4	40	13.19	1.76	better

## Data Availability

The original contributions presented in this study are included in the article. Further inquiries can be directed to the corresponding author.

## Appendix A

Splay, twist, and bend deformations are the three basic forms of deformation seen in the NLC. Each of these three types of deformation can be added to the system’s energy as the Oseen–Frank energy, which is then mixed with the elastic energy. The elastic energy of LC and an electric field, which can be changed by the orientation director, define the overall energy of the system as follows:

(A1) $$F=\int \left(\begin{array}{l} \frac{1}{2}K_{11}{\left(\nabla \cdot n\right)}^{2}+\frac{1}{2}K_{22}(n\cdot {\left(\nabla \times n\right)}^{2})+\frac{1}{2}K_{33}(n\times {\left(\nabla \times n\right)}^{2})\\ -\frac{1}{2}\varepsilon_{0}E^{2}\varepsilon_{r⊥}+\Delta \varepsilon_{r}{\left(\nabla \cdot E\right)}^{2} \end{array}\right)d^{3}r$$

The functional (Ƒ) in Equation (A1) can be minimized using the Euler–Lagrange equation as follows:

(A2) $$\frac{\partial f}{\partial \theta }-\;\frac{d}{dz}\;\cdot \frac{\partial f}{\partial \theta_{z}}$$

and

(A3) $$f=\frac{1}{2}K_{11}{\left(\nabla \cdot n\right)}^{2}+\frac{1}{2}K_{22}(n\cdot {\left(\nabla \times n\right)}^{2})+\frac{1}{2}K_{33}(n\times {\left(\nabla \times n\right)}^{2})-\frac{1}{2}E_{0}E^{2}E_{r⊥}+\Delta E_{r}{\left(n\cdot E\right)}^{2}$$

where *θz* = ə*θ*/ə*z*.

By manipulating the individual terms in Equation (A3), we obtain

(A4) $$f=\frac{1}{2}K_{11}\theta_{z}^{2}\sin^{2}\theta +\frac{1}{2}K_{33}\theta_{z}^{2}\cos^{2}\theta -\frac{1}{2}E_{0}E^{2}E_{r⊥}+\Delta E_{r}\cos^{2}\vartheta$$

Differentiating the resulting terms of Equation (A4), we get

(A5) $$\frac{\partial f}{\partial \theta }=\frac{1}{2}\left(K_{11}-K_{33}\right)\sin2\theta {\left(\frac{\partial \theta }{\partial z}\right)}^{2}+\frac{1}{2}E_{0}\Delta E_{r}E^{2}\sin2\theta$$

(A6) $$\frac{d}{dz}\cdot \frac{\partial f}{\partial \theta_{z}}=\left(K_{11}-K_{33}\right)\sin2\theta {\left(\frac{\partial \theta }{\partial z}\right)}^{2}+\left(K_{11}\sin^{2}\theta -K_{33}\cos^{2}\theta \right)\frac{\partial^{2}\theta }{\partial z^{2}}$$

Substituting Equations (A5) and (A6) in Equation (A2) yields

(A7) $$\frac{1}{2}E_{0}\Delta E_{r}E^{2}\sin2\theta -\frac{1}{2}\left(K_{11}-K_{33}\right)\sin2\theta {\left(\frac{\partial \theta }{\partial z}\right)}^{2}-\left(K_{11}\sin^{2}\theta -K_{33}\cos^{2}\theta \right)\frac{\partial^{2}\theta }{\partial z^{2}}=0$$

Rearranging Equation (A7) yields

(A8) $$\frac{1}{2}\frac{E_{0}\Delta E_{r}E^{2}\sin2\theta }{\left(K_{11}\sin^{2}\theta +K_{33}\cos^{2}\theta \right)}-\frac{1}{2}\frac{\left(K_{11}-K_{33}\right)\sin2\theta }{\left(K_{11}\sin^{2}\theta +K_{33}\cos^{2}\theta \right)}{\left(\frac{\partial \theta }{\partial z}\right)}^{2}-\frac{\partial^{2}\theta }{\partial z^{2}}=0$$

Based on Equation (A8), the Freedericksz transition voltage (Vth) is a voltage with no molecular reorientation that can be reduced, which can be defined as

(A9) $$V_{\mathrm{th}}=\frac{\pi }{h_{1}}\sqrt{\frac{K_{11}}{\;E_{0}\;\Delta E_{r}}}$$

Equation (A8) can be rewritten as

(A10) $$\frac{1}{2}{\left(\frac{V}{V_{th}}\right)}^{2}{\left(\frac{\pi }{h_{1}}\right)}^{2}\frac{\sin2\varphi }{\left(K_{11}\sin^{2}\varphi +K_{33}\cos^{2}\varphi \right)}-\frac{1}{2}\frac{\left(K_{11}-K_{33}\right)\sin2\varphi }{\left(K_{11}\sin^{2}\varphi +K_{33}\cos^{2}\varphi \right)}{\left(\frac{\partial \varphi }{\partial z}\right)}^{2}-\frac{\partial^{2}\varphi }{\partial z^{2}}=0$$
